# Extracorporeal membrane oxygenation with prone position ventilation successfully rescues infantile pertussis: a case report and literature review

**DOI:** 10.1186/s12887-018-1351-0

**Published:** 2018-11-30

**Authors:** Jingyi Shi, Chunxia Wang, Yun Cui, Yucai Zhang

**Affiliations:** 1 Department of Critical Care Medicine, Shanghai Children’s Hospital, Shanghai Jiao Tong University, No.355 Luding Road, Putuo District, Shanghai, 200062 China; 2 Institute of Pediatric Critical Care, Shanghai Jiao Tong University, No.355 Luding Road, Putuo District, Shanghai, 200062 China

**Keywords:** Pertussis, ECMO, Prone position ventilation, Airway spasm, Infant

## Abstract

**Background:**

*Bordetella pertussis* can cause fatal illness with severe acute respiratory distress syndrome (ARDS) and pulmonary hypertension (PHT).

**Case presentation:**

A 6-month-old non-vaccinated boy with *B. pertussis* infection who developed ARDS was treated by extracorporeal membrane oxygenation (ECMO). During his ECMO support stage, sudden occurred decreasing of ECMO flow implied increasing intrathoracic pressure. The airway spasm followed caused sudden drop of ventilator tidal volume as well as poor lung compliance. Prone position ventilation and bundle care were conducted as lung protection ventilator strategy. After 297-h of ECMO support, the patient was weaned off ECMO, and extubated one week later.

**Conclusions:**

In this patient with severe ARDS caused by *Bordetella pertussis*, ECMO was performed for cardiopulmonary support and rescued the infant with severe pertussis. During ECMO support period, prone position ventilation and care bundle nursing strategy contributed to the relief of continuous airway spasm.

**Electronic supplementary material:**

The online version of this article (10.1186/s12887-018-1351-0) contains supplementary material, which is available to authorized users.

## Background

Pertussis, caused by *Bordetella pertussis* infection, is the fifth leading cause of vaccine preventable deaths in children under 5 years of age and remains a public health concern worldwide [1]. For the infant with pertussis, the prognosis is worse if the child develops pneumonia, worsening respiratory failure, PHT and requires mechanical ventilation [2]. For the critical care management of the infant with pertussis, strategies include conventional ventilation, high-frequency oscillatory ventilation, plasmapheresis, inhaled nitric oxygen, leukodepletion, and more recently, extracorporeal membrane oxygenation (ECMO) [2, 3]. Prior reports from the Extracorporeal Life Support Organization (ELSO) Registry demonstrated survival rates of 30% for pertussis patients receiving ECMO support [4, 5], which is rather lower than other ECMO respiratory indications. Recent report based on data from ELSO registry and expanded dataset from individual institutions demonstrated that younger age, vasoactive use, PHT and a rapidly progressive course were independently and significantly associated with higher mortality [3]. The patient with pertussis is characterized by increased mass of leukocytes and high level of pertussis toxin. Pertussis toxin can cause the occlusion of the pulmonary vessels by the increased mass of leukocytes, which leads to PHT, and even includes acute pulmonary vasoconstriction. The refractory airway spasm is the most common clinical manifestation. Until now, there has no report about the effect of recurrent pertussis toxins-induced airway spasm on the management of ECMO in infantile fetal pertussis.

We report the successful outcome of a 6-month-old infant diagnosed as severe pertussis treated by early initiation of ECMO. Both prone position ventilation and care bundle might be considered as key factors of the lifesaving support under ECMO in infant with severe pertussis complicated with recurrent airway spasm.

## Case presentation

**Fig. 1:**
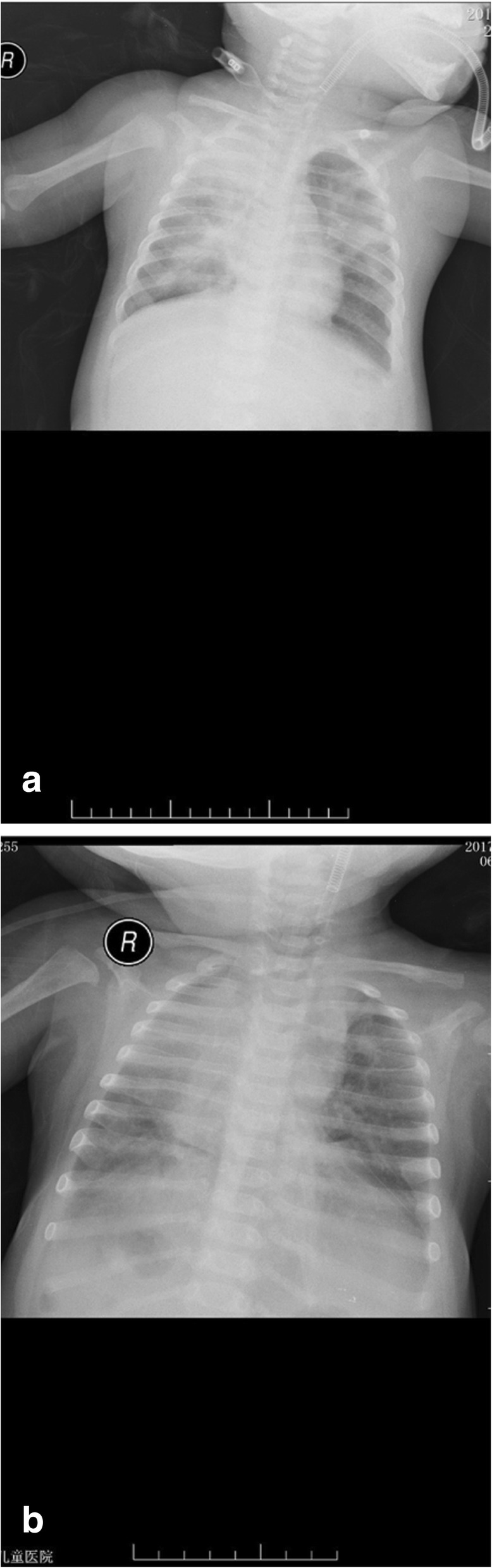
The chest radiograph. (**a**) on admission, (**b**) at 48 h after admission

A 6-month old, 7.5 kg, male baby born at 31 weeks of gestation was admitted to a local hospital with a 20-day history of cough, wheezing, 5-day history of fever and with pleural effusion indicated by chest X-ray. Detection of *Bordetella pertussis* by polymerase chain reaction was positive with the nasopharyngeal specimen. The child was admitted to Pediatric Intensive care unit (PICU) in Shanghai Children’s Hospital with breathless with a temperature of 37.1 °C and heart rate 170 to 190 beats/min, who developed respiratory failure requiring intubated and mechanical ventilation [positive end-expiratory pressure (PEEP) of 6 cmH2O; a pressure support of 18 cmH2O; a respiratory rate (RR) of 25 /min; and a fraction of inspired oxygen of 0.6; Peak inspiratory pressures (PIPs) were between 27 and 29 cmH_2_O]. Meanwhile, laboratory studies revealed the presence of leukocytosis [26,780 white blood cells (WBCs)/μL] with 1 mg/L C-reactive protein (CRP). A chest X-radiograph showed dense opacification of the right upper and right middle lobe and patchy opacification of the left upper lobe (Fig. 1a). Over the next 48 h, despite application of lung protective strategies and a restrictive fluid strategy, the patient deteriorated with worsening lung compliance and hypoxemia, as well as the dense opacification of the right upper and right middle lobe enlarged (Fig.1b). At the same time, the tidal volume decreased from 6 mL/kg to 2.5 mL/kg, and Cdyn (Pulmonary dynamic compliance) decreased from 3.3 (0.44/kg) to 1.2 (0.16/kg). The patient’s PIPs continued to rise to 40 cmH_2_O with a plateau pressure of 31 cmH_2_O, PaO_2_/FiO_2_ dropped to 60 mmHg and oxygen index (OI) raised to 30 lasting for 6 h. Importantly, the echocardiography (performed 3rd day after admission) demonstrated PHT (46 mmHg) with a normal left ventricular function. The diameter of the pulmonary artery was 1 cm, pulmonary artery blood flow was 1.0 m/s, the tricuspid regurgitation flow was 1.5 m/s, the tricuspid annulus was 1.5 cm, and TAPSE was 18. Furthermore, septic shock occurred, norepinephrine (0.3μg/kg.min) and dobutamine (10μg/kg.min) were needed to maintain his blood pressure.

**Fig. 2:**
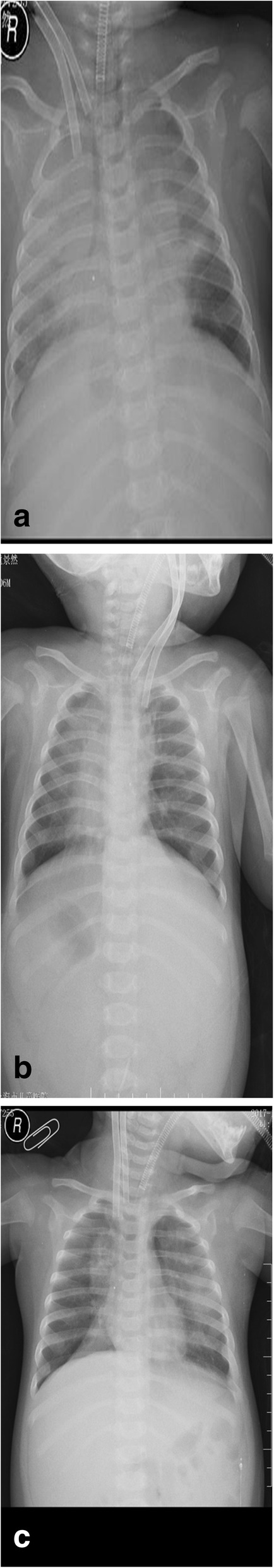
The chest radiograph. (**a**) at the onset of ECMO therapy, (**b**) during prone position ventilation, (**c**) on the 4th day of ECMO support

Considering in anticipation of a rapid hemodynamic collapse resulting from severe PHT in pertussis, the initiation of ECMO was performed with an arterial blood gas noted as pH 7.20, PaCO_2_ 77.9 mmHg and PaO_2_ 60 mmHg. The ECMO support was equipped with a centrifugal pump and an artificial lung was used (Medtronic Bio-Medicus, Minneapolis, MN, USA). The patient inserted cannula through right neck vessels, and was placed on veno-arterial (VA) ECMO support with a pump flow of 500 to 600 mL/min and FiO_2_ of 1.0. The 12-Fr arterial cannula (Bio-Medicus) was placed in the right common carotid artery and the 14-Fr venous cannula (Bio-Medicus) in the right internal jugular vein. (Fig. 2a). During ECMO support, the ventilator setting was mode with SIMV+PS, FiO_2_ 0.4, PIP 25–26 cmH_2_O, PEEP 8-9cmH_2_O, RR 25 rpm, which ensured a 3-5 ml/kg tidal volume. During prone position ventilation, the setting was mode with SIMV+PS, FiO_2_ 0.4, PIP 24-25cmH_2_O, PEEP 8cmH_2_O, RR 20–25 rpm.

During the first 3 days of ECMO support, the tidal volume of this patient was only 2.5 mL/kg, and lung compliance was poor. Prone position ventilation was conducted. A team requires 5 staff members participated. A doctor positioned up to the patient’s head and fixed the catheters, one nurse positioned on each side of the bed to manage the lines and tubes. We firstly placed a blanket around the patient’s arms and turned the patient toward the ventilator. Then, the doctor held the patient’s head and fixed the catheters, while two nurses turned the patient prone. Lastly, we straightened the blanket and adjusted lines and tubes, and placed the patient’s arms in the swimmers position (the arms were positioned up toward the baby’s shoulders). During this process, another doctor was responsible for monitoring the ECMO and a third nurse was responsible for administering drugs. To supine the patient, the process was performed reversely.

There was no change in cannula position shown by X-ray when changing position (Fig. 2a, b), and there was no malfunction of blood access due to bending or dislodgement of the cannula when changing position. At day 4 after ECMO initiation, exhaled tidal volumes were increased to 6 mL/kg and effusion in lung were improved indicated by chest radiographs (Figs. 2c).

**Fig. 3:**
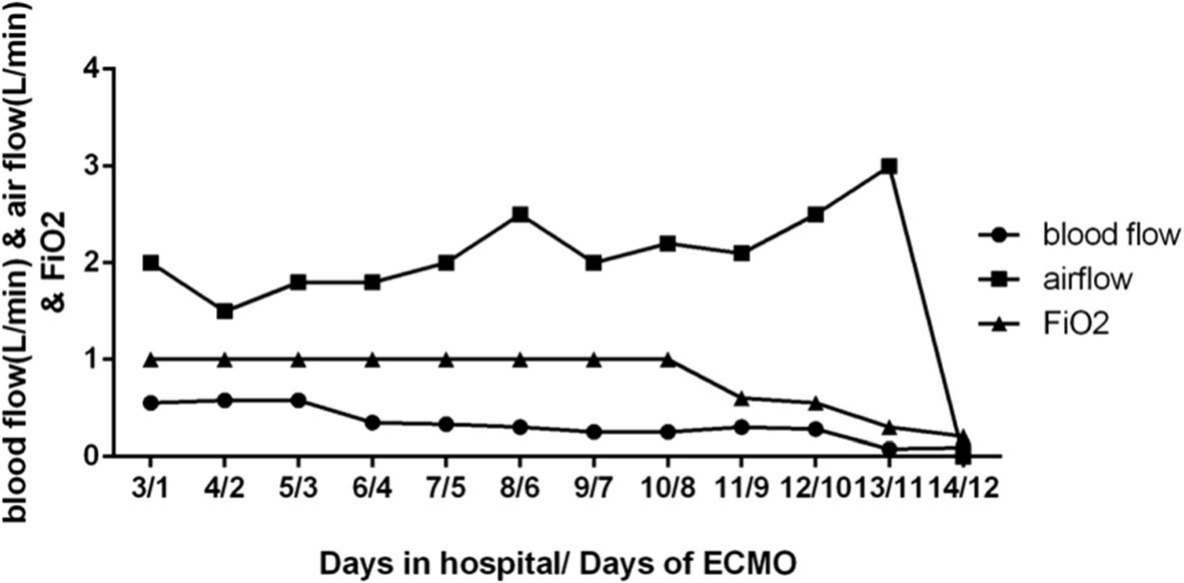
Time course of initiation of ECMO therapy and the ventilator setting

During the first 5 days of ECMO support, the patient was characterized by recurrent attacks of airway spasm lasting for 20s to 2 min each time. Consequently, the airway spasm resulted in sudden declined ECMO flow (0.15 L/min), decreased blood oxygen saturation (80%), and decreased ventilator tidal volume (2 mL/kg). Except for prone position, magnesium sulfate was given and care bundles including hand washing, heightened focus on oral hygiene, closed endotracheal suctioning, prone position ventilation, appropriate sedation, reducing unnecessary stimulus (eg. assess the state of consciousness when put the position), following the nursing sequence of sputum aspiration, diapering and feeding, using full-barrier precautions during the insertion of all catheters were performed to help improving lung compliance and reduce airway spasm. The ECMO flow started around 0.55 L/min and was adjusted according to the hemodynamic status (maintained mean airway pressure [MAP] at 50–60 mmHg). The target blood flow was 80 mL/kg/min, and coagulation profile was monitored through detecting the levels of activated coagulation time (ACT) and activated partial thromboplastin time (aPTT). The values of ACT were detected once 3–4 h and aPTT were detected once per 4–6 h. According to the results of ACT and aPTT, the dose of heparin was regulated to maintain an ACT of 180–220 s or APTT with 1.5–2 fold of normal value. During ECMO therapy, ECMO flow was adjusted to keep the serum ScVO_2_ > 60% and lactate level < 2 mmol/L. The target oxygenation was a normal arterial partial pressure of carbon dioxide (PaCO_2_) and partial pressure of oxygen (PaO_2_) (Fig. 3).

**Fig. 4:**
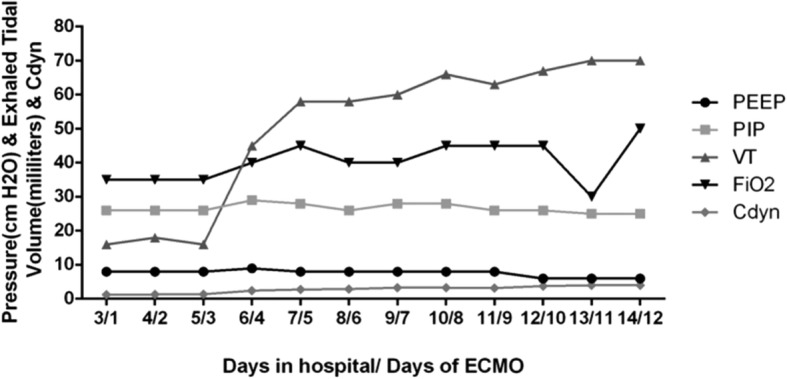
Time course of initiation of ECMO therapy and its effects on pulmonary compliance. After initiation of ECMO therapy, there was sustained recruitment of alveoli evidenced by an increase in the amount of exhaled tidal volume. PEEP, positive end-expiratory pressure; PIP, peak inspiratory pressure, VT, tidal volume; Cdyn, Pulmonary dynamic compliance

**Fig. 5:**
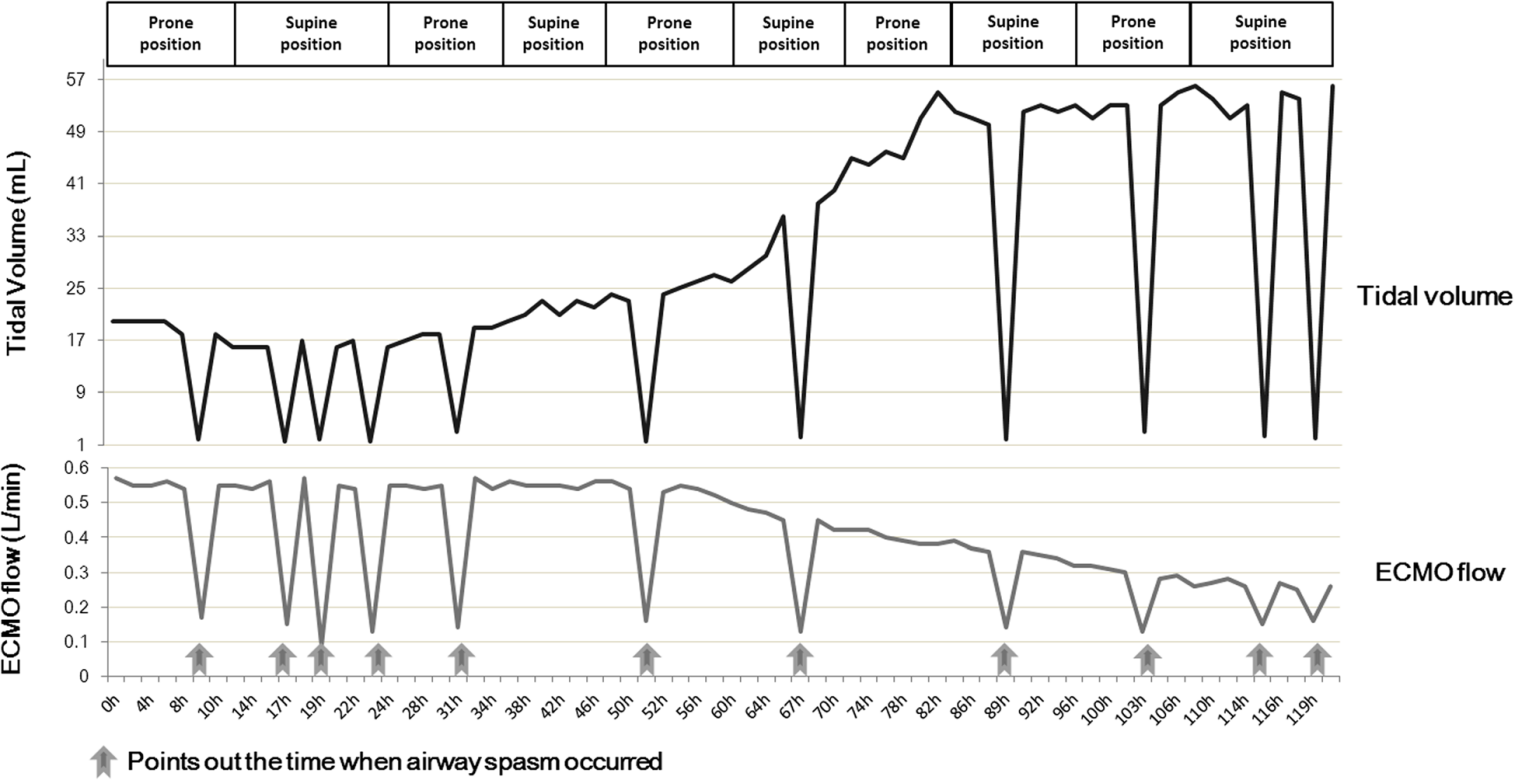
Schematic diagram of timing for the onset and impact of airway spasm on ECMO flow and tidal volume after ECMO initiated, as well as the duration of prone position

From the 6th day of ECMO therapy, the clinical feature of airway spasm was improved. And ventilator setting was gradually reduced. After 297-h of ECMO support, the patient was weaned off V-A ECMO. During the period of ECMO therapy, respiratory mechanical parameters were monitored as shown in Fig. 4. And the ECMO flow dropped with the occurrence of airway spasm, the relationship between tidal volume and ECMO flow were shown in Fig. 5, which represented the onset and impact of airway spasm, as well as the duration of prone position. The enteral nutrition (Infatrini, Nutricia) was conducted through nasogastric tube which provided 80-100 kcal/kg.d.

One week later, the child was successfully extubated. After a 7-day high flow nasal cannula oxygen therapy, he was transferred to the escort ward on 26 days after PICU admission, and eventually discharged with a near normal neurologic examination. The time line of this case can be consulted in the Additional file 1.

## Discussion and conclusions

Patients infected Bordetella pertussis were characterized by cough-associated apnea, cyanosis, PHT and encephalopathy [6]. When these infants with severe respiratory failure is poorly responsive to conventional and alternative therapies, ECMO can be considered as a promising rescue therapy [7], even if pertussis itself implies worse outcome among the indications for ECMO initiation for pediatric respiratory failure [8]. Based on ELSO Registry data, younger age, lower PaO_2_/FiO_2_ ratio, vasoactive use, PHT, and a rapidly progressive course were associated with increased mortality [3].

In this case report, the male child of 6-month-old was successfully recovered by V-A ECMO supporting. Prone position ventilation and care bundle played crucial role in the management of ECMO flow influenced by pertussis toxin-induced airway spasm. Pertussis toxin produces lymphocyte proliferation and results in a hyper viscosity, is thought to be responsible for the leukocytosis observed with pertussis infection [9], and results in a hyper viscosity syndrome that leads to obstruction of the pulmonary arterioles [10]. Here, we describe a 6-month-old boy who developed respiratory failure and septic shock induced by *B. pertussis* infection. The WBC count was 26,780/μL, which was lower than the reported hyperleucocytosis (> 100,000/μL) [11, 12]. It was also reported that young infants infected with *B. pertussis* exhibit prolonged apneic pauses [13]. According to the previous report [14], increased airway pressure decreases the transmural pressure of the right atrium and superior vena cava through lung-heart interaction. Airway spasm causes airway pressure shoot up, then intrathoracic pressure increases and affect the venous return, thus decrease the preload of heart. Another hypothesis is that by previous point of view B. pertussis pneumonia triggers acute pulmonary vasoconstriction resulting in the increased afterload of right heart [15]**.** Given that ECMO flow is affected by the preload and afterload of heart, we speculate that B. pertussis-induced bronchospasm might contribute to a sharp increase of intrathoracic pressure which reduce the venous return, as well as acute pulmonary vasoconstriction reduced ECMO flow through influencing both the preload and afterload of right heart. Importantly, pertussis toxin-induced recurrent airway spasm brought the challenging for the ECMO flow management during EMCO support, which was the specific clinical feature in this case. During the first 5 days of ECMO therapy, recurrent airway spasm resulted in a sudden decline in ECMO flow, decreased blood oxygen saturation, and decreased ventilator tidal volume. It is the first report about the clinical features about pertussis during EMCO supporting according to our knowledge. Magnesium sulfate was used to relieve bronchial spasm, and had some auxiliary functions. Magnesium has an effect on smooth muscle cells, with hypomagnesemia causing contraction and hypermagnesemia causing relaxation. It has been reported that intravenous magnesium sulfate benifit patients with acute severe asthma who do not respond to standard therapeutic medications [16]**.** In addition, a strict following of nursing sequence of sputum aspiration, diapering and feeding, along with appropriate sedative, benefited. Anti-pressure ulcer pads were put under chest and abdomen to preventing possible pressure injury while in prone position. The greatest contribution of care bundle in this patient was minimization of unnecessary stimulus thus gain the time to waiting and recovering from airway spasm. What we concerned most is that frequently sudden drop of ECMO blood flow will cause blood clotting in ECMO circulation, so we monitor both activated clotting time and activated partial thromboplastin time (aPTT) [17]. In our study, the values of ACT and aPTT were correlated well during ECMO therapy. Based on our clinical experience from this case, there was no correlation between the drop of ECMO flow or the shorten of ACT or aPTT, which need further investigation in the future.

Pertussis toxin-induced recurrent airway spasm resulted in decreased arterial oxygen saturation and the increased circulating leukocyte caused by pertussis toxin may restrict pulmonary blood flow, which cause cardiac failure, shock, and acute respiratory distress [15]. Prone position ventilation is an effective method for improving oxygenation in patients with acute respiratory distress syndrome (ARDS) [18, 19]. Importantly, prone positioning can be safely performed and managed among critically ill pediatric patients with ARDS [20–22]. It has been proved that when changed from a supine to prone position, ARDS patients demonstrate a dramatic redistribution of CT lung densities because of re-expansion of previously atelectatic posterior regions. After perfusion improves in these previously hypoxic, vasoconstricted posterior lung regions when turn to prone position, ventilation/ perfusion improves [23]**.** When the patient ventilated prone position, the central blood volume increased by shift of splanchnic blood volume to the thorax, which could induce pulmonary vascular recruitment, then airway spasm relief, thus help to prevent reductions in ECMO flow. On the other hand, prone position may facilitate sputum drainage. So we assume that prone position ventilation does not directly relief airway spasm, but it improves ventilation/ perfusion, facilitate sputum drainage, thus improves lung compliance. The effect of prone position on oxygenation-lung compliance of pediatric ARDS patients may be in doubt. From the research in adult ARDS patients, both low tidal volumes and increased proning duration contribute to a lower mortality in ARDS patients, and the effects were marked in the subgroup in which the duration of prone positioning was more than 10 h/session [24–26]. Recent study indicated that prone positioning was performed for 4 h, every 8 h, for 10 days in a 17-day-old infant with severe pertussis under ECMO support [10]. Thus, for pediatric ARDS patients, the duration and efficacy of prone position need to be verified through more clinical trials. In this case, prone ventilation proved to be effective for improving compliance indicated by significantly increased low Cdyn after supine to prone and then prone to supine position. And cannula-related complications such as accidental removal or dislodgement of a central venous catheter, tracheal tube and cannula for extracorporeal circulation did not happened during prone positioning. These results suggested that prone positioning is a safe and effective procedure in patients with severe pertussis receiving extracorporeal circulation.

There were some limitation in this case report. We did not continuously detect the WBC counts during ECMO support. Since the patient was born premature, we are now unable to report on the long-term outcome of the patient’s neurodevelopment. These need further investigation in cased with severe pertussis treated by ECMO supporting.

From this case review, we speculated that ECMO management is challenging in patients with pertussis contributing to the high mortality of these patients under ECMO support. Prone position ventilation contributes to better oxygenation and lung compliance. And detailed care bundle is essential for patients with pertussis challenged by recurrent airway spasm.

## Additional file

Additional file 1: Time line of this case. (DOCX 128 kb)

## Abbreviations

ACT: Activated clotting time
aPTT: Activated partial thromboplastin time
ARDS: Acute respiratory distress syndrome
Cdyn: Pulmonary dynamic compliance
CRP: C-reactive protein
ECMO: Extracorporeal membrane oxygenation
ELSO: Extracorporeal Life Support Organization
MAP: Mean airway pressure
OI: Oxygen index
PaCO_2_: Arterial partial pressure of carbon dioxide
PaO_2_: Partial pressure of oxygen
PEEP: Positive end-expiratory pressure
PHT: PHT
PICU: Pediatric Intensive care unit
PIP: Peak inspiratory pressure
RR: Respiratory rate
ScVO_2_: Central venous oxygen saturation
WBC: White blood cells
