# Supplementary material for: Extracorporeal membrane oxygenation with prone position ventilation successfully rescues infantile pertussis: a case report and literature review
Source: BMC Pediatr. 2018 Nov 30;18:377. doi: 10.1186/s12887-018-1351-0 (PMC6267074; doi:10.1186/s12887-018-1351-0)
Supplement: Supplementary file 1 — Time line of this case. (DOCX 128 kb) [file 12887_2018_1351_MOESM1_ESM.docx]

6-month male baby born of 31 weeks of gestation, non-vaccinated


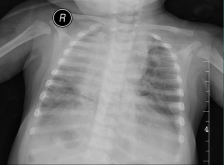


2017.9.26

11:00

nasopharyngeal specimen *B.pertussis* positive by PCR

2017.9.25

Admitted to PICU for respiratory failure

2017.9.23

First presented as cough and wheezing to the pediatric clinic

2017.9.4

Presentation of the patient, diagnose confirmed, and pathogen deteched

Treatment of the patient

Severe ARDS developed and ECMO supported. Prone position ventilation conducted.


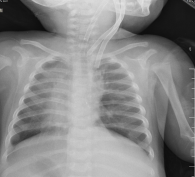


Weaned off ECMO.

2017.10.7

20:00

Extubated

2017.10.14

12:00

Transferred to the escort ward

2017.10.18
